# Cucurbitacin L 2-O-****β****-Glucoside Demonstrates Apoptogenesis in Colon Adenocarcinoma Cells (HT-29): Involvement of Reactive Oxygen and Nitrogen Species Regulation

**DOI:** 10.1155/2012/490136

**Published:** 2012-02-26

**Authors:** Siddig Ibrahim Abdelwahab, Loiy Elsir Ahmed Hassan, Amin M. S. Abdul Majid, Sakina M. Ahmed Yagi, Syam Mohan, Manal Mohamed Elhassan Taha, Syahida Ahmad, Cheah Shiau Chuen, Putri Narrima, Mohd Mustafa Rais, Suvitha Syam, Bushra Abdulkarim Moharam, A. Hamid A. Hadi

**Affiliations:** ^1^Department of Pharmacy, Faculty of Medicine, University of Malaya, 50603 Kuala Lumpur, Malaysia; ^2^Department of Botany, Faculty of Science & Technology, Omdurman Islamic University, P.O. Box 383, Omdurman, Sudan; ^3^Department of Pharmacology, School of Pharmaceutical Sciences, Universiti Sains Malaysia, Pulau Pinang, 11800 Minden, Malaysia; ^4^Department of Botany, Faculty of Science, University of Khartoum, P.O. Box 321, Khartoum, Sudan; ^5^Centre of Natural Products and Drug Discovery (CENAR), Department of Pharmacology, Faculty of Medicine, University of Malaya, 50603 Kuala Lumpur, Malaysia; ^6^Department of Biochemistry, Faculty of Biotechnology, Universiti Putra Malaysia, UPM, 43400 Serdang, Selangor, Malaysia; ^7^School of Medicine, Faculty of Medical Sciences, UCSI University, 56000 Kuala Lumpur, Malaysia; ^8^Department of Chemistry, Faculty of Science, University of Malaya, 50603 Kuala Lumpur, Malaysia

## Abstract

Emerging evidence suggests that reactive oxygen (ROS) and nitrogen (RNS) species can contribute to diverse signalling pathways of inflammatory and tumour cells. Cucurbitacins are a group of highly oxygenated triterpenes. Many plants used in folk medicine to treat cancer have been found to contain cucurbitacins displaying potentially important anti-inflammatory actions. The current study was designed to investigate the anti-ROS and -RNS effects of cucurbitacin L 2-O-*β*-glucoside (CLG) and the role of these signaling factors in the apoptogenic effects of CLG on human colon cancer cells (HT-29). This natural cucurbitacin was isolated purely from *Citrullus lanatus* var. *citroides* (Cucurbitaceae). The results revealed that CLG was cytotoxic to HT-29. CLG increased significantly (*P* < 0.05) RNA and protein levels of caspase-3 in HT-29 cells when verified using a colorimetric assay and realtime qPCR, respectively. The results showed that lipopolysaccharide/interferon-gamma (LPS/INF-*γ*) increased nitrous oxide (NO) production inR AW264.7macrophages, whereas N(G)-nitro-L-argininemethyl ester (L-NAME) and CLG curtailed it. This compound did not reveal any cytotoxicity on RAW264.7 macrophages and human normal liver cells (WRL-68) when tested using the MTT assay. Findings of ferric reducing antioxidant power (FRAP) and oxygen radical absorption capacity (ORAC) assays demonstrate the antioxidant properties of CLG. The apoptogenic property of CLG on HT-29 cells is thus related to inhibition of reactive nitrogen and oxygen reactive species and the triggering of caspase-3-regulated apoptosis.

## 1. Introduction

The attractive association between chronic inflammation and cancer has been a fertile field for the growth of biomedical research. In particular, the development of colon cancer is a distinctive situation in which inflammatory conditions such as ulcerative colitis increase the risk of cancer by 20-fold [1, 2]. The presence of certain inflammation markers, such as the C-reactive protein circulating in the blood, is correlated with an increased risk of colon cancer [3]. In addition, overexpression of proinflammatory enzymes, such as inducible nitric oxide synthase and cyclooxygenase-2, has been reported in human colon cancer and in an azoxymethane-induced colon cancer model in rats [4, 5]. More importantly, selective inhibitors of these inflammatory genes are effective in inhibiting experimental colon cancer of rodents [6, 7].

Epithelial cells express reactive nitrogen and oxygen species (free radicals) in response to inflammatory cytokines and the bacterial endotoxins. The initiation by NADPH (nicotinamide adenine dinucleotide phosphate) oxidase is always required for the production of free radicals. These free radicals were activated upon translocation of several cytosolic proteins to the membrane-bound complex carrying cytochrome c. Moreover, the activation of NADPH oxidase can be caused by microbial products such as lipopolysaccharide and lipoproteins, by IFN-*γ* (interferon-gamma), by IL-8 (interleukin-8), or by IgG binding to Fc-receptors. The primary product of the reaction catalyzed by the NADPH oxidase is superoxide (O_2_^−^), which can be converted to H_2_O_2_ by superoxide dismutase (SOD), to hydroxyl radicals (^.^OH) and hydroxyl anions (OH^−^) by the iron-catalyzed Haber-Weiss reaction, or after dismutation to H_2_O_2_, to hypochlorous acid (HOCl) and chloramines by myeloperoxidase [8]. Depending on the cell type, various downstream signalling pathways are also involved in the transcriptional regulation of ROS and RNS. This phenomenon of cellular inflammatory response also takes place on other types of cells. This is also tested experimentally in cancer and inflammation models. Epithelial cells have been implicated in many of these experiments, as they are directly involved with the inflammatory response [9–12]. One of the important roles of these cells is the production of various cytokines, reactive oxygen and nitrogen species, growth factors, and chemokines as a response to activation signals such as chemical mediators, cytokines, and bacterial lipopolysaccharide [13]. Although the bioactive molecules produced by macrophages have valuable outcomes in inflammation, these molecules were also shown to have unfavorable and damaging effects. Hence, the modulation of these products provides a target for controlling inflammatory and malignant diseases [3].

Wild melon (*Citrullus lanatus* var. *citroides*; belonging to the Cucurbitaceae family) is a low climbing, hairy, and annual plant. Cucurbit plants were used actively as traditional herbal remedies for various diseases including cancer [14–17]. Cucurbit plants have demonstrated anti-inflammatory, antitumor, liver protective, and immune-regulatory activities. This family is also known to contain several bioactive compounds such as cucurbitacins, triterpenes, sterols, and alkaloids. Plants containing cucurbitacins were early recognized in folk medicine to have biological value. Many plants used in folk medicine to treat inflammatory conditions have been found to contain triterpenoids displaying potentially important anti-inflammatory actions, in different *in vivo* and *in vitro* assays, as well as inhibition of hydrolytic enzyme activity and lipid peroxidation [18]. In this regard, the root extract of *Wilbrandia ebracteata *(Cucurbitaceae), a plant commonly used in Brazil to treat rheumatic disease, was reported to contain several cucurbitacins [19]. There are limited studies on the chemopreventive effect and the mechanism of action of CLG. Therefore, the present study attempts to understand the inhibitory effects of CLG on reactive nitrogen and oxygen species as an explanation for the apoptogenic effects of this natural cucurbitacin in colon cancer cells (HT-29). Based on our studies, the anti-inflammatory and antioxidant properties of CLG may be regarded as a key attribute for its role against colon tumorigenesis.

## 2. Materials and Methods

### 2.1. Cell Lines and Reagents

All cell lines were obtained from American type culture collection (ATCC). Dulbecco's Modified Eagle Medium (DMEM) both with and without phenol red, phosphate buffered saline and Hanks' balanced salt solution (HBSS), 3-(4,5-dimethylthiazol-2-yl)-2,5-diphenyltetrazoliumbromide (MTT), phosphate buffered saline (PBS), and Griess reagent were from Invitrogen (Carlsbad, USA). Fetal bovine serum (FBS), LPS from *E. coli* serotype 0111 : B4, L-NAME, dimethylsulfoxide (DMSO), and sodium nitrite were obtained from Sigma (St. Louis, USA). IFN*γ* was from BD Biosciences (New Jersey, USA). All other chemicals and reagents used were of HPLC grade.

### 2.2. Isolation of Cucurbitacin L 2-O-*β*-Glucoside

*Citrullus latanus *var*. citriode* was collected from AL-Musawwarat area, Northern Sudan, on February 2008. The voucher specimen was identified by Dr. Wai'l S. Abdalla, a senior botanist at the herbarium of Medicinal and Aromatic Plants Research Institute (MAPRI), Khartoum, Sudan, where the specimen was also deposited and coded with CL2-8. CLG was isolated from the dried fruit pulp according to the method described earlier [20]. The structure of this compound was established by spectroscopic methods and by comparison with the previous reported works [20–22]. Purity of the compound was found to be 98.5% using LC/MS.

### 2.3. 3-(4,5-Dimethylthiazol-2-yl)-2,5-Diphenyltetrazolium Bromide Cell Viability Assay on Normal and Cancer Cells

All the cells were maintained in a 37°C incubator with 5% CO_2_ saturation. Human hepatocellular carcinoma cells (HepG2), colon adenocarcinoma cells (HT-29), and normal hepatic cells (WRL-68) were maintained in Dulbecco's modified Eagle's medium (DMEM), whereas non-small-cell lung cancer cells (A549) and prostate adenocarcinoma cells (PC3) were maintained in RPMI-1640 medium. Both media were supplemented with 10% fetal bovine serum (FBS), 100 units/mL penicillin, and 0.1 mg/mL streptomycin.

Different cell types from above were used to determine the cytotoxic effects of CLG and paclitaxel on cancer and normal cells using the MTT assay. For measurement of cell viability, cells were seeded at a density of 1 × 10^5^ cells/mL in a 96-well plate and incubated for 24 h at 37°C and 5% CO_2_. Cells were treated and incubated for 24 h. After 24 h, MTT solution at 2 mg/mL was added for 4 h. Absorbance was measured at 570 nm. Results were expressed as a percentage of control giving percentage cell viability after 24 h exposure to test agents. The potency of cell growth inhibition for the tested compounds was expressed as an EC_50_ value, defined as the concentration that caused a 50% loss of cell growth. Viability was defined as the ratio (expressed as a percentage) of absorbance of treated cells to untreated cells.

### 2.4. Chromatin Condensation Assay

For detection of apoptotic cells, apoptotic nuclear morphology was observed by staining with Hoechst 33342. Cells at a density 1 × 10^5^ cells/mL were seeded on a 96-well culture plate. Cells were treated with IC_50_ of CLG and incubated for 24 and 48 h. After treatment, cells were washed with PBS and stained with Hoechst 33342 (1 mM), and plates were analyzed using the ArrayScan HCS system (Cellomics, PA, USA).

### 2.5. Colorimetric Assays of Caspase-3

The colorimetric protease assay of caspase-3 provides a simple and convenient means for quantifying the enzyme activity that recognizes the amino acid sequence, DEVD (A synthetic tetrapeptide, (Asp-Glue-Val-Asp), which is the upstream amino acid sequence of the caspase-3 (cleavage site), coupled with p-nitroanilide, which is released upon substrate cleavage. This assay was performed using the commercial kit Apo*Target* (Code: KHZ0022: BioSource International, Inc., USA). 1 × 10^5^ cells/mL were treated with IC_50_ of CLG and incubated for 24 and 48 h while untreated cells acted as control. The cells were lysed by the addition of 50 *μ*L of chilled Cell Lysis Buffer and incubated on ice for 10 min. The resulting cell lysate was centrifuged for 1 min at 10,000 ×g, and the supernatant was collected. Fifty microliters of 2X Reaction Buffer (containing 10 mM DTT) was added to each sample. Then 5 *μ*L of DEVD-*p*NA (caspase-3 substrate) was added and incubated in the dark at 37°C for 1 h. At the end of the incubation period, the samples were read at 405 nm in a microplate reader (TECAN, SunriseTM, Männedorf, Switzerland). Data was presented as optical density (405 nm; mean ± SD).

### 2.6. Real-Time Polymerase Chain Reaction

Total RNA was extracted using RNeasy Mini Kit following the manufacturer's instructions (Qiagen, Germantown, Maryland, USA). RNA concentrations were quantified using a spectrophotometer (Smart Spec, Bio-Rad). RNA quality and integrity were determined via the A260/A280 ratio and agarose gels electrophoresis, respectively. cDNA was synthesized with Revert Aid H Minus M-muLV reverse transcriptase (Biometra, Goettingen, Germany). Real-time RT-PCR was performed using an ABI 7700 Prism Sequence Detection System and TaqMan primer probes (Applied Biosystems, Foster City, CA). The total reaction volume was 20 *μ*L: 2 *μ*L cDNA, 10 *μ*L SYBR Premix ExTaq, 0.4 *μ*L of each primer (10 *μ*M; caspase-3 primer; sense, 5′-TTAATAAAGGTATCCATGGAGAACACT-3′; antisense, 5′-TTAGTGATAAAAATAGAGTTCTTTTGTGAG-3′), and 7.2 *μ*L ultrapure water. Cycle parameters were as follows: activation at 95°C for 30 s, 40 cycles of denaturation at 95°C for 5 s, and then annealing and extension at 60°C for 30 s. Glyceraldehyde-3-phosphate dehydrogenase gene (GAPDH) was used as an internal control for each sample. The primers for GAPDH were 5′-GGTGGTCTCCTCTGACTTCAACA-3′ (sense) and 5′-GTTGCTGTAGCCAAATTCGTTGT-3′ (antisense). PCR products were detected using gel electrophoresis.

### 2.7. Effect of CLG on Nitric Oxide Production

#### 2.7.1. Cell Culture and Stimulation

The murine monocytic macrophages cell line (RAW 264.7) was maintained in DMEM supplemented with 10% FBS, 4.5 g/L glucose, sodium pyruvate (1 mM), L-glutamine (2 mM), streptomycin (50 *μ*g/mL), and penicillin (50 U/mL) at 37°C and 5% CO_2_. Cells at confluency of 80–90% were centrifuged at 120 ×g at 4°C for 10 min, and cell concentration was adjusted to (2 × 10^6^) cells/mL, whereby the cell viability was always more than 90%, as determined by trypan blue exclusion. A total of 50 *μ*L of cell suspension was seeded into a tissue culture grade 96-well plate (4 × 10^5^ cells/well) and incubated for 2 h at 37°C, 5% CO_2_ for cell attachment. Then, the cells were stimulated by using 100 U/mL of IFN-*γ* and 5 *μ*g/mL of LPS with or without the presence of CLG and L-NAME (250 *μ*M) tested at the final volume of 100 *μ*L/well. DMSO was used as vehicle, where the final concentration of DMSO was maintained at 0.1% of all cultures. Cells were further incubated at 37°C, 5% CO_2_ for 20 h. The culture supernatant was subjected to Griess assay for nitrite determination, and the cells remaining in the well were tested for cell viability assay by using MTT reagent.

#### 2.7.2. Griess Assay

To evaluate the inhibitory activity of CLG and L-NAME on nitric oxide (NO) production, culture media was assayed using Griess reaction [23]. Briefly, an equal volume of Griess reagent (1% sulphanilamide and 0.1% N-(l-naphthyl)-ethylene diaminedihydrochloride, dissolved in 2.5% H_3_PO_4_) was mixed with the culture supernatant, and colour development was measured at 550 nm using a microplate reader (SpectraMax Plus, Molecular Devices Inc., Sunnyvale, CA, USA). The amount of nitrite in the culture supernatant was calculated from a standard curve (0–100 *μ*M) of sodium nitrite freshly prepared in deionized water. Percentage of the NO inhibition was calculated by using nitrate level of IFN-*γ*/LPS-induced group as the control,

(1) $$\begin{array}{cc} & \text{NO}\;\text{inhibitory}\;\left(\%\right)\\ & \;\;=\frac{{\left[{{\text{NO}}_{2}}^{-}\right]}_{\text{control}}-{\left[{{\text{NO}}_{2}}^{-}\right]}_{\text{sample}}}{{\left[{{\text{NO}}_{2}}^{-}\right]}_{\text{control}}}\times 100\text{\%}. \end{array}$$

#### 2.7.3. Cell Viability of RAW 264.7 Macrophage

The cytotoxicity of CLG on cultured cells was determined by assaying the reduction of MTT reagents to formazan salts [24]. After removing the supernatant, the MTT reagents (5 mg/mL, dissolved in sterile PBS, pH 7.0) were added into each well. The cells remaining were incubated at 37°C for 4 h, and the formazan salts formed were dissolved by adding 100 *μ*L of 100% DMSO in each well. The absorbance was then measured at 570 nm using SpectraMax Plus microplate reader (Molecular Devices, USA). The percentage of cell viability was calculated by using the cell viability of IFN-*γ*/LPS-induced group as the control,

(2) $$\begin{array}{cc} & \text{cell}\;\text{viability}\;\left(\%\right)\\ & \;\;=\frac{{\text{OD}}_{\text{control}}-\;{\text{OD}}_{\text{sample}}}{{\text{OD}}_{\text{control}}}\times 100\text{\%}. \end{array}$$

### 2.8. Antioxidant Capacity of CLG

#### 2.8.1. Oxygen Radical Absorbance Capacity

The oxygen radical absorbance capacity (ORAC) assay was done to test the antioxidant capacity of CLG based on the procedure described earlier with slight modifications [25]. Briefly 175 *μ*L of the sample/blank was dissolved with PBS at concentrations of 160 *μ*g/mL, pH 7.4, 75 mM, and serial dilutions for the Trolox standards were prepared accordingly. ORAC assay was performed in a 96-well black microplate with 25 *μ*L of samples/standard/positive control and 150 *μ*L of fluorescence sodium salt solution, followed by 25 *μ*L of 2,20-azobis (2-amidinopropane) dihydrochloride (AAPH) solution after 45-minute incubation at 37°C (200 *μ*L total well volume). Fluorescence was recorded until it reached zero (excitation wavelength at 485 nm, while emission wavelength at 535 nm) in a fluorescence spectrophotometer (Perkin-Elmer LS 55), equipped with an automatic thermostatic autocell holder at 37°C. The positive control was Quercetin, and the negative control was blank solvent/PBS. Data were collected every 2 min for a duration of 2 h. Results were calculated using the differences of areas under the fluorescein decay curve (AUC) between the blank and the sample and are expressed as Trolox equivalents.

#### 2.8.2. Ferric Reducing/Antioxidant Power Assay

The determination of the total antioxidant activity (FRAP assay) of CLG is a modified method of Benzie and Strain [26]. The stock solutions included 300 mM acetate buffer (3.1 g C_2_H_3_NaO_2_ *·*3H_2_O and 16 mL C_2_H_4_O_2_), pH 3.6, 10 mM TPTZ (2, 4, 6-tripyridyl-s-triazine) solution in 40 mMHCl, and 20 mM FeCl_3_ *·*_6_H_2_O solution. The fresh working solution was prepared by mixing 25 mL acetate buffer, 2.5 mL TPTZ, and 2.5 mL FeCl_3_ *·*_6_H_2_O. The temperature of the solution was raised to 37°C before use. CLG (10 *μ*L) was allowed to react with 300 *μ*L of the FRAP solution in the dark. Readings of the coloured product (ferrous tripyridyltriazine complex) were taken at 593 nm. The standard curve was linear between 100 and 1000 *μ*M FeSO_4_. Results are expressed in *μ*M Fe(II)/g dry mass and compared with that of ascorbic acid and quercetin.

### 2.9. Statistical Analysis

The data obtained was statistically analyzed using one-way ANOVA. This was followed by Dunnett's or Tukey's post hoc tests when the ANOVA produced significant results. All data were expressed as the mean ± S.E.M. The tests were performed using GraphPad Software version 5.01 (GraphPad Software Inc., San Diego, CA). Differences are considered significant when *P* < 0.05.

## 3. Results

### 3.1. Cytotoxicity of CLG

The panel of cell lines used in this study were human hepatocellular carcinoma cells (HepG2), prostate adenocarcinoma cells (PC3), colon adenocarcinoma cells (HT-29), non-small-cell lung cancer cells (A549), and normal hepatic cells (WRL-68). The cytotoxicity assay (MTT) performed in this study revealed that the CLG had demonstrated a dose-depended effect on HT-29. The EC_50_ value (the sample concentration reducing the absorbance of treated cells by 50% with respect to untreated cells) of CLG on the viability of HT-29 has been determined to be 79.76 ± 2.34 *μ*g/mL (Table 1). All cells were observed to be resistant to CLG. Fortunately, CLG did not produce any toxic effects on normal hepatic cells until the concentration of 200 *μ*g/mL. Paclitaxel showed high indiscriminate cytotoxicity to all cells used in this study.

### 3.2. Chromatin Condensation Assay

**Figure 1:**
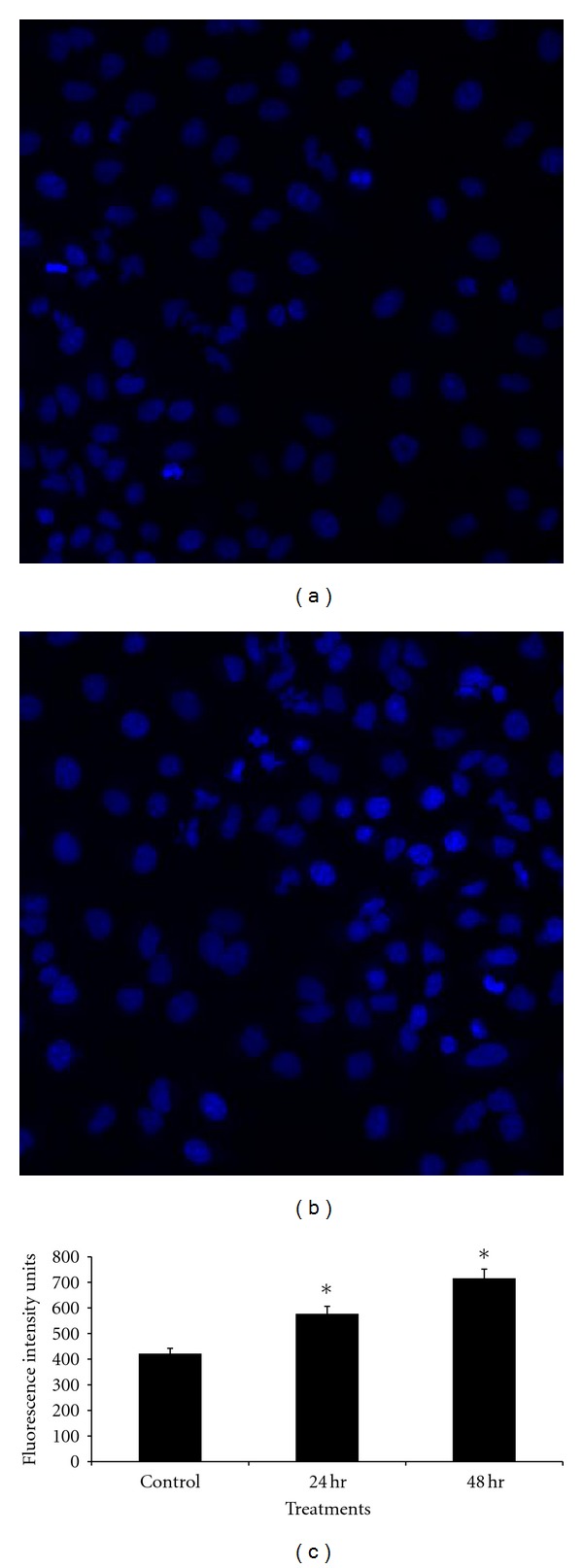
Fluorescent photomicrographs of cells stained Hoechst 33342 being treated with with CLG (IC_50_) for 24 and 48 h. (a) Control, (b) chromatin condensation in the nucleus (48 h), and (c) quantitative analysis of apoptosis (total nuclear intensity). Statistical significance is expressed as *, *P* < 0.05.

We then investigated apoptosis in cells that had been incubated with CLG using the DNA staining dyes Hoechst 33342. After a 24-hour incubation with CGL, the percentage of apoptotic cells was significantly increased compared with the control (medium alone) (*P* < 0.05). Following incubation of cells with CGL for 72 h, the apoptosis was also significantly increased (*P* < 0.05). The increased percentage of fluorescent intensity is the direct representation of chromatin condensation in the nucleus (Figures 1(a) and 1(b)).  Figure 1(c) summarizes the results of CGL-dependent apoptosis, that is, the percentage of apoptosis above control levels.

### 3.3. Colorimetric Assay of Caspase-3

**Figure 2:**
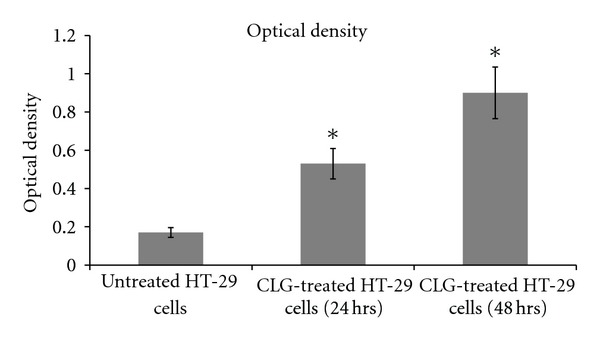
The colorimetric assay of caspase-3 in colon adenocarcinoma cells (HT-29) treated and untreated with CLG (IC_50_) for 24 and 48 h. ANOVA showed a significant difference (*P < 0.05) between treated and untreated cells in the activity of caspase-3.

Since HT-29 has shown remarkable sensitivity to CLG, an *in vitro* colorimetric assay of caspase-3 was conducted to assess apoptosis between control and treated cells. As shown in Figure 2, CLG significantly (independent *t*-test, *P* < 0.05) stimulated caspase-3, the hallmark enzyme of apoptosis. The level of this enzyme is higher in treated HT-29 cells as compared to nontreated cells; this concludes that CLG induces cell death towards human colon cancer cells, HT-29.

### 3.4. Gene Expression

**Figure 3:**
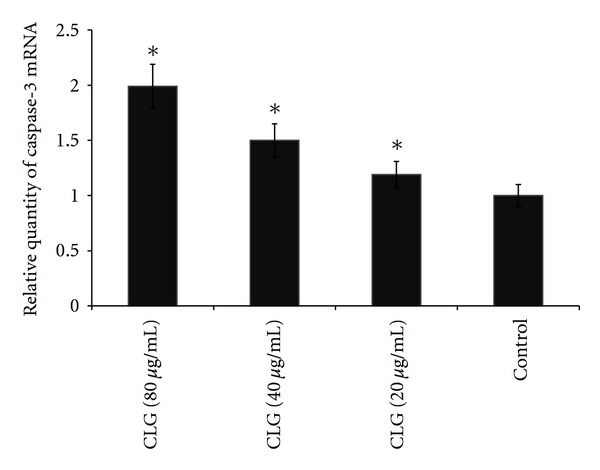
Caspase-3 mRNA expression in HT-29. Cells were treated with CLG and then incubated with for 24 h. After incubation, cells were harvested and used real-time RTPCR analysis. Normalization relative to GAPDH was performed. Results presented in bar graph are the means ± SD of three independent experiments. The statistical significance is expressed as *, *P* < 0.05.

To examine whether the apoptotic effect of CLG is due to intervention with caspase-3-regulated apoptosis, mRNAs of HT-29 cells were studied using qRT-PCR. Caspase-3 mRNA level was significantly increased in cells exposed to CLG compared to levels in control cells (Figure 3).

### 3.5. Effect of CLG on Nitric Oxide from RAW 264.7 Macrophage

**Figure 4:**
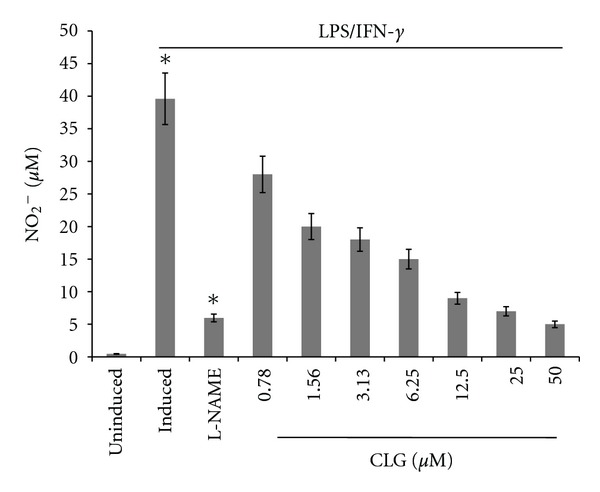
The effects of CLG on NO production in RAW264.7 cells: cells were pretreated with the indicated concentrations of CLG, or the NO inhibitor L-NAME. The cells were stimulated with LPS and INF or were left untreated. The nitric oxide was measured using Griess assay. Data is representative of three independent experiments and was analysed using one-way ANOVA. The inhibitory effect of CLG was significantly different from stimulated cells.

**Figure 5:**
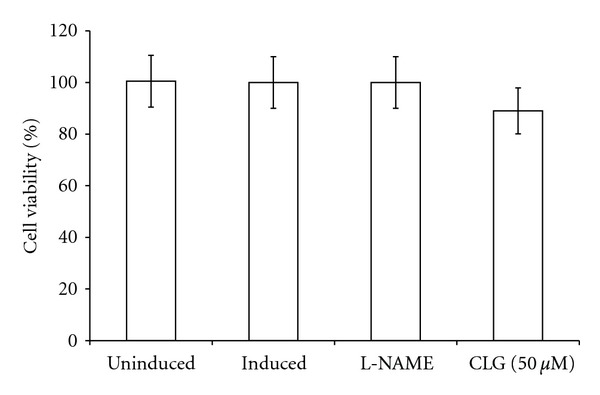
The effects of CLG on RAW264.7 cells' viability: cells were pretreated with the indicated concentrations of CLG or were left untreated. Data is the average of three independent experiments (±SD) and was analyzed using one-way ANOVA. The statistical significance is expressed as *, *P* < 0.05.

Stimulation with LPS and IFN-*γ* led to fortyfold increase in nitrite concentrations in the cell supernatant with a concentration of 39.76 ± 1.2 *μ*M as compared to the basal level of 1.0 ± 0.05 *μ*M in untreated cells (Figure 4). NO release was effectively inhibited by CLG with an IC_50_ of 26.5 ± 1.81 *μ*M. Following 20 h of treatment and stimulation, CLG caused a 90% inhibition of NO at 75 *μ*M, without affecting the cell viability (Figure 5). L-NAME, as a positive control anti-NO drug, caused an 84% inhibition of NO at 250 *μ*M.

### 3.6. ORAC Antioxidant Activity Assay

To evaluate the antioxidant capacity of CLG, ORAC assay was used, and the potency of this natural compound was compared with the positive control, quercetin. The area under the curve (AUC) was calculated for the CLG, trolox, and quercetin. ORAC results are shown in Table 1. CLG displays considerable antioxidant activity. Whereby, this compound at 20 *μ*g/mL is equivalent to a concentration of 82.5 ± 0.53 *μ*M of trolox. Quercetin at 5 *μ*g/mL is equivalent to a concentration of 160.32 ± 2.75 *μ*M of Trolox.

### 3.7. FRAP Assay

**Figure 6:**
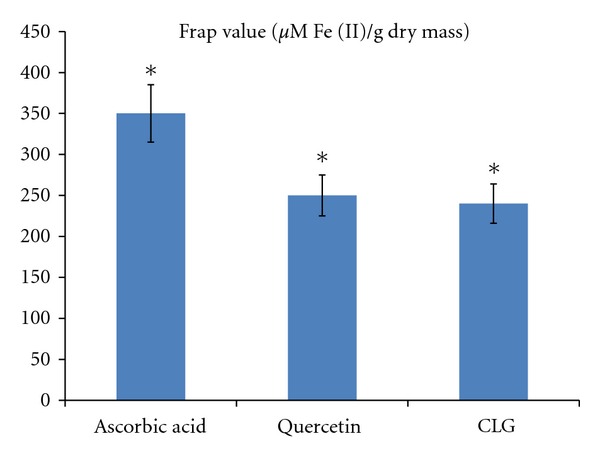
Ferric reducing/antioxidant power assay. Results are expressed in *μ*M Fe(II)/g dry mass and compared with that of ascorbic acid and quercetin. ANOVA. The statistical significance is expressed as *, *P* < 0.05.

The total antioxidant activity was determined using FRAP assay. This assay measures the reduction of ferric iron (Fe^3+^) to ferrous iron (Fe^2+^) in the presence of antioxidants, which are reductants with half-reaction reduction potentials above Fe^3+^/Fe^2+^. On treatment, CLG exhibited a significant FRAP value, with a 241±12.5 *μ*mol/L, while the positive control used in this study exhibited a value of  350 ± 9.5  and  251 ± 5.7  for ascorbic acid and quercetin, respectively (Figure 6).

## 4. Discussion

The cucurbitacins are of great interest because of the wide range of biological activities they exhibit in plants and animals [20]. In the present study, we investigated for the first time the apoptogenic effects of cucurbitacin L 2-O-*β*-glucoside (CLG) on cancer cell (HT-29) and the involvement of reactive oxygen and nitrogen species regulation. Our results indicate that CLG is more cytoselective to colon cancer cells than the other tested cancer cells. The inhibitory effects of CLG on RNS and ROS are suggested to be the mechanism of apoptogenic effect of this natural compound. Based on our study, the anti-inflammatory and antioxidant properties of CLG may be regarded as a key attribute for its role against colon tumorigenesis.

Nitric oxide (NO) is a free radical gas with important immune, cardiovascular, and neurological second messenger functions that is implicated in sepsis, cancer, and inflammation. This molecule is synthesized from the amino acid L-arginine by a family of enzymes, the nitric oxide synthases (NOSs). NO in colonic mucosa is susceptible to manipulation by proinflammatory cytokines [27–30]. The obtained results suggest that the compound has dose-dependent anti-inflammatory activities related with their inhibition of NO production in macrophages without affecting the viability of these cells. Our results are in line with previous findings which showed that cucurbitacin compounds are able to inhibit the production of NO [27, 28].

Anticolorectal cancer agents have an inhibitory effect on nitrite production in colonic mucosa and could play an anti-inflammatory role in intestinal inflammation and malignancies [31]. NO has also been demonstrated to be involved in the inhibition of apoptosis in a number of cell types including leukocytes, hepatocytes, trophoblasts, and endothelial cells such as HT-29 [32]. Generally, the antiapoptotic effects of NO can be mediated through a number of mechanisms such as the nitrosylation and inactivation of many of the caspases including caspase 3. As shown in Figure 2, CLG significantly (independent *t*-test, *P* < 0.05) stimulated caspase-3 (protein and gene), the hallmark enzyme of apoptosis. Previous reports have shown that the ability of cucurbitacin D to induce apoptosis is related with its ability to activate caspase-3 in cancer cells [33]. Earlier, we also observed the onset of apoptosis marked by the nuclear fragmentation using Hoechst 33342 dye. Both the morphological assessment of changes in apoptotic nuclei and the activation of caspase-3 induced by CGL in this study had validated the presence of involvement of apoptosis in the cell death occurred.

Although the mechanism by which cucurbitacin glycosides act as antioxidants is not well understood, it is generally believed that the beneficial properties of cucurbitacin glycosides are due to their ability to directly interact with reactive oxygen species [20]. The current study shows for the first time that CLG is an effective antioxidant when tested using both ORAC and FRAP assays. These results suggest that CLG could be used to suppress oxygen cellular metabolites. Previous data have demonstrated that the cucurbitacin E glycoside exhibits antioxidant properties, probably through the involvement of a direct scavenging effect on several free radicals [20]. On the other hand, NO can freely interact with oxygen metabolites to yield nitrosating species, and the formation of nitrosamines may be important in the development of colorectal cancer in ulcerative colitis. High levels of nitrosamines have been demonstrated in rectal dialysates of patients with active inflammatory bowel disease [12]. Therefore, the obtained results warrant further research for the clinical application of CLG as chemopreventive agents for advanced ulcerative colitis patients.

Selective apoptogenesis of potential anticancer drugs have been reported previously for various chemical entities with the aid of molecular modelling and structure activity relationship (SAR) studies [34–36]. Previous SAR study with five cucurbitacin analogues led to a highly selective STAT3, a proliferation protein, and cucurbitacin Q; a highly selective inhibitor of JAK2 activation, cucurbitacin A; three dual inhibitors, cucurbitacin I, E, and B. From the chemical point of view, these findings indicate that addition of a single hydroxyl group to carbon 11 of the cucurbitacins results in loss of anti-STAT3 activity, whereas a simple conversion of a carbon 3 carbonyl to a hydroxyl leads to loss of anti-JAK2 activity [37].

In summary, the present study showed that the apoptogenic effects of cucurbitacin L 2-O-*β*-glucoside on the tested cancer cells is selectively directed to HT-29. The suppressive effects of CLG on reactive oxygen and nitrogen species regulation are very much correlated to cell death induced in HT-29. Based on our studies, the anti-inflammatory and antioxidant effects of CLG may be regarded as a key attribute for its role against chronic ulcerative colitis and colon tumorigenesis.

## 5. Conclusion

Cucurbitacin is one of the constituents of plants used in folk medicine. Till date, there was no study reporting the apoptogenic effects of CLG on human colon cancer cells (HT-29). The current study shows for the first time that the apoptogenic property of CLG on HT-29 cells is related to the inhibition of reactive nitrogen and oxygen species eventually leading to caspase-3-mediated apoptosis. At present, the pathways related to apoptosis are being studied in our lab.

## Figures and Tables

**Table 1:** Effect of CLG and paclitaxel on different cells type expressed as EC_50_ values in MTT assay.

Cell line	Tissue of human origin	Compound EC_50_ ± SD (*μ*g/mL)	
		Cucurbitacin L 2-O-*β*-Glucoside	Paclitaxel
A549	Non-small-cell lung cancer	>200	5.81 ± 1.03
PC-3	Human prostate carcinoma	>200	0.08 ± 0.03
HepG2	Hepatocellular carcinoma	>200	1.18 ± 0.24
HT-29	Colon adenocarcinoma	79.76 ± 2.34	0.06 ± 0.02
WRL-68	Normal hepatic cells	>200	0.10 ± 0.05

## References

[1] Taha MME, Abdul AB, Abdullah R, Ibrahim TAT, Abdelwahab SI, Mohan S (2010). Potential chemoprevention of diethylnitrosamine-initiated and 2-acetylaminofluorene-promoted hepatocarcinogenesis by zerumbone from the rhizomes of the subtropical ginger (*Zingiber zerumbet*). *Chemico-Biological Interactions*.

[2] Björkman M, Klingen I, Birch ANE (2011). Phytochemicals of Brassicaceae in plant protection and human health—influences of climate, environment and agronomic practice. *Phytochemistry*.

[3] Il’yasova D, Colbert LH, Harris TB (2005). Circulating levels of inflammatory markers and cancer risk in the health aging and body composition cohort. *Cancer Epidemiology Biomarkers and Prevention*.

[4] Rao CV, Indranie C, Simi B, Manning PT, Connor JR, Reddy BS (2002). Chemopreventive properties of a selective inducible nitric oxide synthase inhibitor in colon carcinogenesis, administered alone or in combination with celecoxib, a selective cyclooxygenase-2 inhibitor. *Cancer Research*.

[5] Takahashi M, Mutoh M, Kawamori T, Sugimura T, Wakabayashi K (2000). Altered expression of *β*-catenin, inducible nitric oxide synthase and cyclooxygenase-2 in azoxymethane-induced rat colon carcinogenesis. *Carcinogenesis*.

[6] Rao CV, Kawamori T, Hamid R, Reddy BS (1999). Chemoprevention of colonic aberrant crypt foci by an inducible nitric oxide synthase-selective inhibitor. *Carcinogenesis*.

[7] Luceri C, Caderni G, Sanna A, Dolara P (2002). Red wine and black tea polyphenols modulate the expression of cycloxygenase-2, inducible nitric oxide synthase and glutathione-related enzymes in azoxymethane-induced F344 rat colon tumors. *Journal of Nutrition*.

[8] Bogdan C, Röllinghoff M, Diefenbach A (2000). Reactive oxygen and reactive nitrogen intermediates in innate and specific immunity. *Current Opinion in Immunology*.

[9] Gloire G, Legrand-Poels S, Piette J (2006). NF-*κ*B activation by reactive oxygen species: fifteen years later. *Biochemical Pharmacology*.

[10] Tian J, Kim SF, Hester L, Snyder SH (2008). S-nitrosylation/activation of COX-2 mediates NMDA neurotoxicity. *Proceedings of the National Academy of Sciences of the United States of America*.

[11] Wimalawansa SJ (2008). Nitric oxide: new evidence for novel therapeutic indications. *Expert Opinion on Pharmacotherapy*.

[12] Peyrot F, Ducrocq C (2008). Potential role of tryptophan derivatives in stress responses characterized by the generation of reactive oxygen and nitrogen species. *Journal of Pineal Research*.

[13] Ibrahim MY, Abdul ABH, Ibrahim TAT, AbdelWahab SI, Elhassan MM, Mohan S (2010). Attenuation of cisplatin-induced nephrotoxicity in rats using zerumbone. *African Journal of Biotechnology*.

[14] Okoli BE (1984). Wild and cultivated cucurbits in Nigeria. *Economic Botany*.

[15] Murakami A, Jiwajinda S, Koshimizu K, Ohigashi H (1995). Screening for *in vitro* anti-tumor promoting activities of edible plants from Thailand. *Cancer Letters*.

[16] Jayaprakasam B, Seeram NP, Nair MG (2003). Anticancer and antiinflammatory activities of cucurbitacins from *Cucurbita andreana*. *Cancer Letters*.

[17] Attard E, Brincat MP, Cuschieri A (2005). Immunomodulatory activity of cucurbitacin E isolated from *Ecballium elaterium*. *Fitoterapia*.

[18] Siqueira JM, Peters RR, Gazola AC (2007). Anti-inflammatory effects of a triterpenoid isolated from *Wilbrandia ebracteata* Cogn. *Life Sciences*.

[19] Peters RR, Farias MR, Ribeiro-do-Valle RM (1997). Anti-inflammatory and analgesic effects of cucurbitacins from *Wilbrandia ebracteata*. *Planta Medica*.

[20] Tannin-Spitz T, Bergman M, Grossman S (2007). Cucurbitacin glucosides: antioxidant and free-radical scavenging activities. *Biochemical and Biophysical Research Communications*.

[21] Tannin-Spitz T, Grossman S, Dovrat S, Gottlieb HE, Bergman M (2007). Growth inhibitory activity of cucurbitacin glucosides isolated from *Citrullus colocynthis* on human breast cancer cells. *Biochemical Pharmacology*.

[22] Mata R, Castañeda P, Camacho MDR, Delgado G (1988). Chemical studies on Mexican plants used in traditional medicine, V. Cucurbitacin glucosides from *Cigarrilla mexicana*. *Journal of Natural Products*.

[23] Granger DL, Taintor RR, Boockvar KS, Hibbs JB (1996). Measurement of nitrate and nitrite in biological samples using nitrate reductase and Griess reaction. *Methods in Enzymology*.

[24] Mosmann T (1983). Rapid colorimetric assay for cellular growth and survival: application to proliferation and cytotoxicity assays. *Journal of Immunological Methods*.

[25] Cao G, Alessio HM, Cutler RG (1993). Oxygen-radical absorbance capacity assay for antioxidants. *Free Radical Biology and Medicine*.

[26] Benzie IFF, Strain JJ (1998). Ferric reducing/antioxidant power assay: direct measure of total antioxidant activity of biological fluids and modified version for simultaneous measurement of total antioxidant power and ascorbic acid concentration. *Methods in Enzymology*.

[27] Park CS, Lim H, Han KJ (2004). Inhibition of nitric oxide generation by 23, 24-dihydrocucurbitacin D in mouse peritoneal macrophages. *Journal of Pharmacology and Experimental Therapeutics*.

[28] Annegowda HV, Mordi MN, Ramanathan S, Mansor SM (2010). Analgesic and antioxidant properties of ethanolic extract of *Terminalia catappa* L. leaves. *International Journal of Pharmacology*.

[29] Huang TY, Chu HC, Lin YL (2009). Minocycline attenuates experimental colitis in mice by blocking expression of inducible nitric oxide synthase and matrix metalloproteinases. *Toxicology and Applied Pharmacology*.

[30] Tanaka T, Shimizu M, Kohno H (2001). Chemoprevention of azoxymethane-induced rat aberrant crypt foci by dietary zerumbone isolated from *Zingiber zerumbet*. *Life Sciences*.

[31] Bonavida B, Baritaki S, Huerta-Yepez S, Vega MI, Jazirehi AR, Berenson J (2010). Nitric oxide donors are a new class of anti-cancer therapeutics for the reversal of resistance and inhibition of metastasis. *Nitric Oxide (NO) and Cancer*.

[32] Ma Q, Wang Z, Zhang M (2010). Targeting the L-arginine-nitric oxide pathway for cancer treatment. *Current Pharmaceutical Design*.

[33] Takahashi N, Yoshida Y, Sugiura T, Matsuno K, Fujino A, Yamashita U (2009). Cucurbitacin D isolated from *Trichosanthes kirilowii* induces apoptosis in human hepatocellular carcinoma cells *in vitro*. *International Immunopharmacology*.

[34] Ciardiello F, Caputo R, Bianco R (2000). Antitumor effect and potentiation of cytotoxic drugs activity in human cancer cells by ZD-1839 (Iressa), an epidermal growth factor receptor- selective tyrosine kinase inhibitor. *Clinical Cancer Research*.

[35] Fulda S, Debatin KM (2005). Sensitization for anticancer drug-induced apoptosis by betulinic acid. *Neoplasia*.

[36] Nebbioso A, Clarke N, Voltz E (2005). Tumor-selective action of HDAC inhibitors involves TRAIL induction in acute myeloid leukemia cells. *Nature Medicine*.

[37] Sun J, Blaskovich MA, Jove R, Livingston SK, Coppola D, Sebti SM (2005). Cucurbitacin Q: a selective STAT3 activation inhibitor with potent antitumor activity. *Oncogene*.

